# Pore Structure and Limit Pressure of Gas Slippage Effect in Tight Sandstone

**DOI:** 10.1155/2013/572140

**Published:** 2013-11-28

**Authors:** Lijun You, Kunlin Xue, Yili Kang, Yi Liao, Lie Kong

**Affiliations:** State Key Laboratory of Oil and Gas Reservoir Geology and Exploitation, Southwest Petroleum University, Chengdu 610500, China

## Abstract

Gas slip effect is an important mechanism that the gas flow is different from liquid flow in porous media. It is generally considered that the lower the permeability in porous media is, the more severe slip effect of gas flow will be. We design and then carry out experiments with the increase of backpressure at the outlet of the core samples based on the definition of gas slip effect and in view of different levels of permeability of tight sandstone reservoir. This study inspects a limit pressure of the gas slip effect in tight sandstones and analyzes the characteristic parameter of capillary pressure curves. The experimental results indicate that gas slip effect can be eliminated when the backpressure reaches a limit pressure. When the backpressure exceeds the limit pressure, the measured gas permeability is a relatively stable value whose range is less than 3% for a given core sample. It is also found that the limit pressure increases with the decreasing in permeability and has close relation with pore structure of the core samples. The results have an important influence on correlation study on gas flow in porous medium, and are beneficial to reduce the workload of laboratory experiment.

## 1. Introduction

With the development of oil and gas exploration technology, tight gas reservoirs, the most realistic unconventional reservoirs, play and will continually play an increasingly vital role in gas reserves and supply [1]. According to the third resource assessment, tight sandstone gas resources in China are about 20 × 10^12^ m^3^. Tight sandstone reservoirs face the huge difficulty of the exploitation because of slim throat, low porosity, low permeability, high content of clay mineral, and high capillary pressure. Gas slip effect, a phenomenon that will occur when gas flowing through a thin capillary tube or a fine porous medium, controls gas flow behavior and severely affects the ability of gas flow in tight sandstone gas reservoir. During this process, the velocity of gas in velocity layer in the immediate vicinity of the solid walls of the capillary or porous medium is not zero, which will cause an increase in gas flow rate in porous media [2–5].

Klinkenberg (1941) was the first to introduce the concept of gas slip effect into gas permeability measurement; the mathematical expression was given as [5]

(1) $$\begin{array}{c} K_{a}=K_{\infty }\left(1+\frac{b}{p_{m}}\right). \end{array}$$

 
*K*_*a*_ is gas permeability, *μ*m^2^.
 
*K*_*∞*_ is Klinkenberg permeability, *μ*m^2^.
 
*P*_*m*_ is mean pressure, MPa.
 
*b* is gas slip factor, affected by pressure, temperature, pore structure of porous medium, and type of gas. The expression was given as
  (2) $$\begin{array}{c} b=\frac{4C\lambda p_{m}}{r}. \end{array}$$
 
*λ* is mean free path of gas molecules, mm.
 
*r* is radius of a capillary or a pore, mm.
 
*C* is constant.

It is indicated by (2) that gas slip factor is inversely proportional to radius of capillary.

According to Darcy's law, the expression of gas permeability is given as

(3) $$\begin{array}{c} K_{a}=\frac{2Qp_{0}\mu L}{A\left(p_{1}^{2}-p_{2}^{2}\right)}\times 10^{-1}. \end{array}$$

 
*Q* is volumetric flow rate, cm^3^/s.
 
*μ* is dynamic viscosity of the fluid, mPa·s.
 
*A* is cross-sectional area, cm^2^.
 
*L* is length of core sample, cm.

Transforming (3)

(4) $$\begin{array}{c} Q\mu =\frac{5K_{a}A}{p_{0}}\times \frac{p_{1}^{2}-p_{2}^{2}}{L}. \end{array}$$

From (4), the relationship between *Qμ* and (*p*_1_^2^ − *p*_2_^2^)/*L* is a straight line with increasing backpressure. (*p*_1_^2^ − *p*_2_^2^) is composed of two items. One means the pressure drop across core sample (*p*_1_ − *p*_2_) and the other is double the pore pressures of core sample (*p*_1_ + *p*_2_). When *Qµ* and (*p*_1_^2^ − *p*_2_^2^)/*L* are linear relationships and the gradient is a stable value, gas permeability is equal to Klinkenberg permeability. And the gas slip effect would be reduced with the increasing of inlet pressure [6].

Both permeability and gas reservoir pressure determine the extent of slippage effect impacting volumetric flow rate [7–9]. The lower the permeability and gas pressure are, the more prominent gas slip effect would be [10]. The influence factors of gas slip effect include permeability, pore pressure, and water saturation. Gas slip effect would be prominent when the permeability is less than 0.1 × 10^−3^ *μ*m^2^ and the pore pressure is a low value, while the specific boundaries of water saturation are not clear [11]. Gas slip factor is related to the pore structure [12].

Slippage effect affects gas production. In laboratory, gas permeability is usually measured at a succession of pressures to obtain the Klinkenberg permeability by correcting because of Slippage effect. Laboratory working is increased. Equation (1) suggests that gas permeability is equal to Klinkenberg permeability when *b*/*p*_*m*_ = 0. Some researchers indicated that gas slip effect can be prevented by increasing pore pressure of high permeable core samples, but the study about tight sandstone is rare, and the results of tight sandstone are very different. Until Now there is no terminology to describe this phenomenon. In the past, some researchers exerted a big backpressure by rule of thumb to reduce Klinkenberg effect, which increases the pressure-bearing demand of experimental cardholder.

When gas permeability was close to Klinkenberg permeability by improving mean pressure to cause *b*/*p*_*m*_ to approach to zero, we define the pore pressure or backpressure at the outlet of the core sample as limit pressure.

If we know the limit pressure, we can measure permeability by exerting a backpressure which is equal to or a little greater than limit pressure to mitigate slippage effect on experimental results, such as the effect of gas velocity on gas permeability due to fine migration [13].

The impetus for this work was a concern that finding the relation between limit pressure of eliminating gas slippage effect and pore structure parameters can help obtain the limit pressure of specific pore structure rock.

## 2. Experimental Samples and Procedures

### 2.1. Core Samples

**Figure 1:**
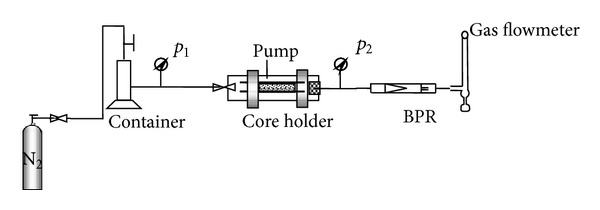
Schematic diagram of the experimental apparatus.

In this study, the tight sandstone core samples, from Permian in Upper Paleozoic in Ordos basin, involve four permeability levels (<0.1 × 10^−3^ *μ*m^2^, (0.1~0.3) × 10^−3^ *μ*m^2^, (0.3~1) × 10^−3^ *μ*m^2^, and >1 × 10^−3^ *μ*m^2^). Nitrogen is regarded as displacing medium. The schematic diagram of the experimental apparatus is shown in Figure 1. It mainly consists of a high pressure core holder, a high pressure nitrogen cylinder, a high pressure pump, a backpressure regulator (BPR), and a gas flowmeter.

### 2.2. Procedure

**Figure 2:**
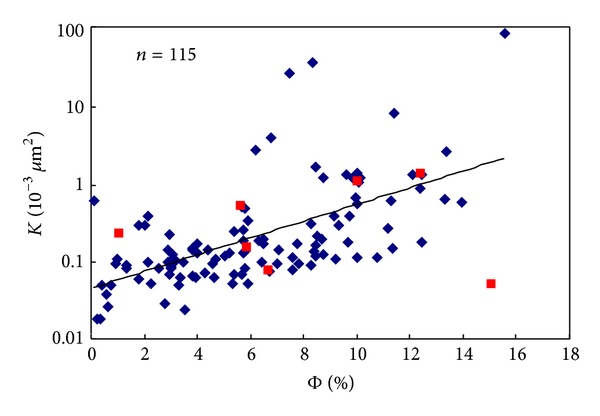
Relationships between porosity and permeability at 3 MPa for core samples.

(1) Seven samples of four permeability levels (<0.1 × 10^−3^ *μ*m^2^, (0.1~0.3) × 10^−3^ *μ*m^2^, (0.3~1) × 10^−3^ *μ*m^2^, and >1 × 10^−3^ *μ*m^2^) are selected in the experiments. SS-1 is an outcrop sample that is different from others. Before conducting the porosity and permeability test, the core samples in this work are dried for more than 48 hours at 60°C. The basic parameters of samples are listed in Table 1.  Figure 2 shows the relationship between porosity and permeability for core samples. The red ones are the samples in the experiments. (2) After a core sample is installed into the core holder, a confining pressure of 7 MPa is applied. Before flow tests, the core sample is needed to stay at this confining pressure for at least four hours to make sure that the stress equilibrium is reached. To start a test, the outlet pressure is set at a designed backpressure. In this test, the backpressure increases from 0 MPa and its differential ranges from 0.1 MPa to 0.2 MPa. (3) When the backpressure is fixed, the inlet pressure is increased by using the regulator of the nitrogen cylinder. The pressure difference between inlet and outlet is 0.5 MPa, 1.0 MPa, 1.5 MPa, 2.0 MPa, and 2.5 MPa, respectively. Once a steady flow is reached, the gas flow rate at different pressures is recorded and the permeability is calculated. (4) Increase backpressure and repeat step (3). (5) Analyze the experiment data and illustrate *Qμ* versus (*p*_1_^2^ − *p*_2_^2^)/*L* plots and *K*_*a*_ versus 1/*p*_*m*_ plots.

## 3. Results

### 3.1. Relationship between the Product of Flow Rate and Viscosity and Pressure Gradient

**Figure 3:**
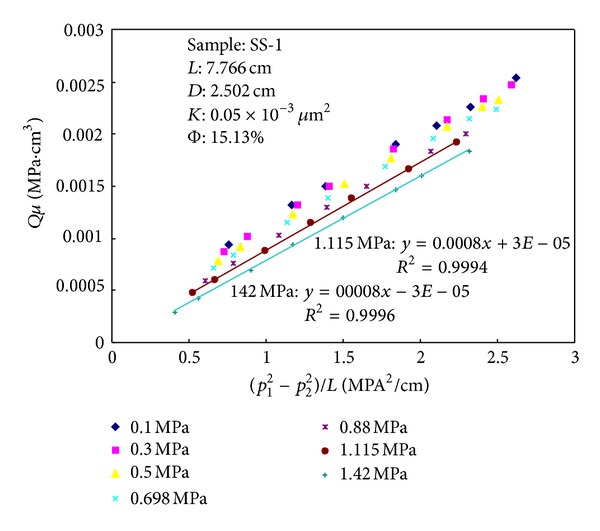
Relationships between (*p*_1_^2^ − *p*_2_^2^)/*L* and *Qμ* at various backpressures for sample SS-1.

**Figure 4:**
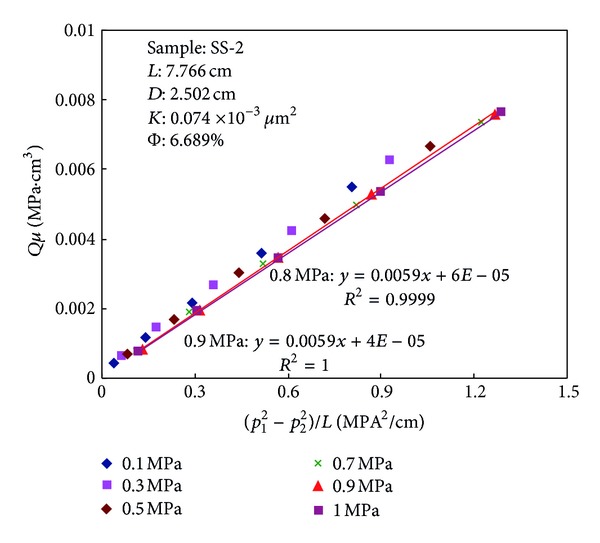
Relationships between (*p*_1_^2^ − *p*_2_^2^)/*L* and *Qμ* at various backpressures for sample SS-2.

*Qμ* and (*p*_1_^2^ − *p*_2_^2^)/*L* are plotted in Figures 3 and 4. It can be seen from Figures 3 and 4 that with the increasing of backpressure the slopes of the curves gradually reduce and the intercepts gradually approach to zero. When the backpressure reaches a specific level, the slope of the curve does not change with the pressure and intercept is equal to zero. The regression coefficient *R* ^2^ is more than 0.999. Equation (4) demonstrates that the gas slip effect can be eliminated when the permeability does not change with pressure. As shown in Figure 4, the relationship between *Qμ* and (*p*_1_^2^ − *p*_2_^2^)/*L* is linear relation and the intercept is equal to zero when the backpressure at outlet reaches 0.9 MPa. The regression coefficient *R* ^2^ = 0.9999. When the outlet pressure of the sample exceeds 1 MPa, the curve is also fit for the law.

### 3.2. Relationship between Permeability and Reciprocal of Mean Pressure

**Figure 5:**
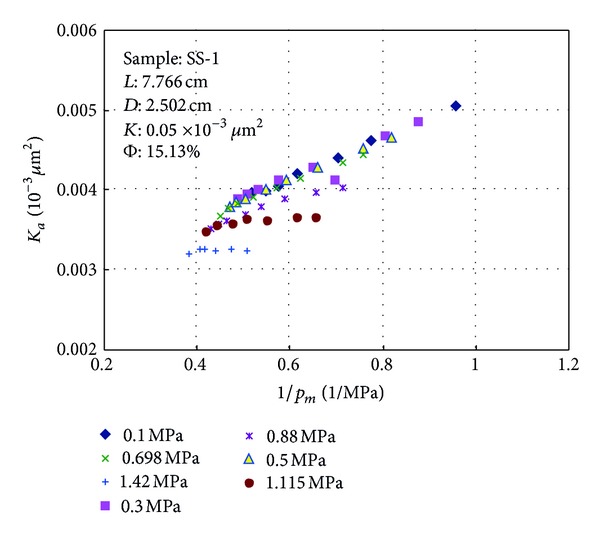
Relationships between 1/*p*_*m*_ and *K*_*a*_ at various backpressures for sample SS-1.

**Figure 6:**
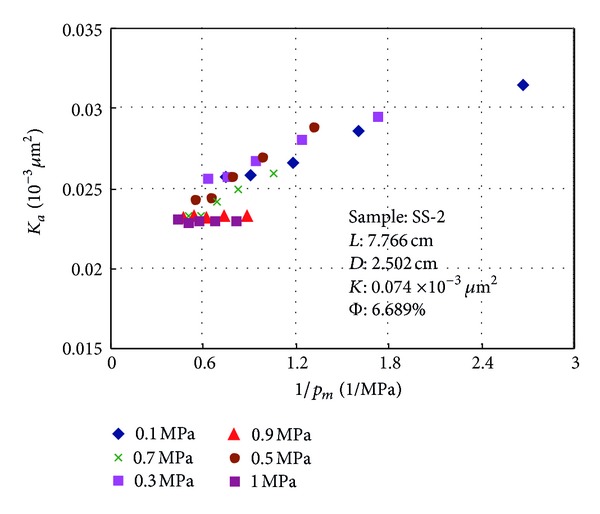
Relationships between 1/*p*_*m*_ and *K*_*a*_ at various backpressures for sample SS-2.

Relationship between *K*_*a*_ and 1/*p*_*m*_ is presented in Figures 5 and 6. Figures 5 and 6 show that slip effect is obvious and permeability decreases with the increasing of mean pressure when the outlet pressure is atmospheric pressure. When the outlet pressure increases to a certain level, the relationship between *K*_*a*_ and 1/*p*_*m*_ is nearly horizontal and the gas permeability tested at different pressure drops is almost a stable value whose range is less than 3% and slip factor is less than 0.05 for a given sample (Table 2). The backpressure at outlet of the core sample is defined as limit pressure and the permeability is equal to liquid permeability.

Gas slip factor *b* for sample SS-2 at different backpressure calculated from (1) is shown in Table 3. It can be seen from Table 3 that gas slip factor significantly reduced the increasing of backpressure and gas slip factor is less than 0.05 when the backpressure exceeds 0.9 MPa. At this case, gas slip effect can be eliminated.

## 4. Discussion

*(1) Gas Flow State in Tight Sandstone under Backpressure.* Microstructure of tight sandstone is complicated, thereby Darcy's law only is not enough to describe the process of gas flow in micropore [14]. Gas flows in the different porous medium. Based on different mean free paths of gas molecules, the gas flow in micropore has different regions [15].

Knudsen (1934) introduced the concept of Knudsen number Kn, as is given by

(5) $$\begin{array}{c} \text{Kn}=\frac{\bar{\lambda }}{D}, \end{array}$$

where $\bar{\lambda }$ is mean free path of gas molecules and *D* is pore throat diameter

(6) $$\begin{array}{c} \bar{\lambda }=\frac{KT}{\sqrt{2}\pi d^{2}P}. \end{array}$$

Gas flow condition in micropore medium is decided by petrophysical property of the medium and mean free path of gas molecules [16, 17]. From the study of Liepmann, Stahl, and Kaviany et al. gas flow in tight sandstone is divided into three regions according to Knudsen number. It includes flow region, transition flow region, and viscous flow region.

Based on the results of Roy et al., gas flow in tight sandstone reservoir is divided by Knudsen number [18].

Ortega and Aguilera (2012) indicated that *R*_35_ in tight sandstone was the throat radius when the saturation was 35%. It can be defined as mean throat radius. Empirical formula is given as [19]

(7) $$\begin{array}{c} \log R_{35}=0.732+0.588\log K-0.864\log\phi . \end{array}$$

Based on the porosity and permeability of core samples, *R*_35_ for SS-3 was calculated by (7), as shown in Table 2.

When the throat radius *R*_35_ = 0.382 *μ*m, the Knudsen number Kn at different outlet pressure was calculated by (5) and (6) as shown in Table 5.

From Table 4, Knudsen number Kn is greater than 0.001 and the gas slip effect is obvious when the pressure at outlet of core samples is atmospheric pressure. As the outlet pressure exceeds 0.6 MPa, Knudsen number Kn is less than 0.001 and the slip effect is negligible, which belongs to Darcy flow. Thus the gas slip effect can be neglected when the backpressure at outlet equals or exceeds the limit pressure.

**Figure 7:**
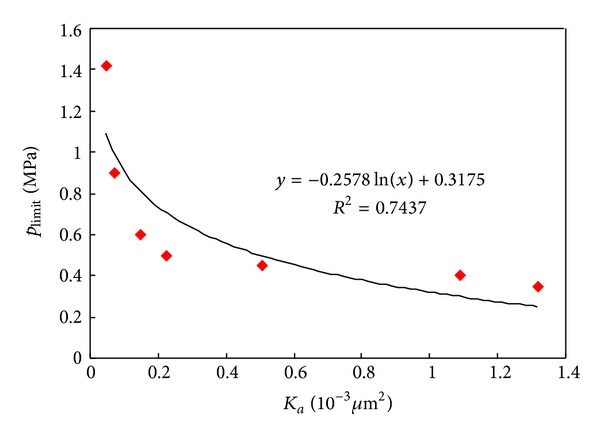
Relationships between *K*_*a*_ and *p*_limit_ for tight core samples.

**Figure 8:**
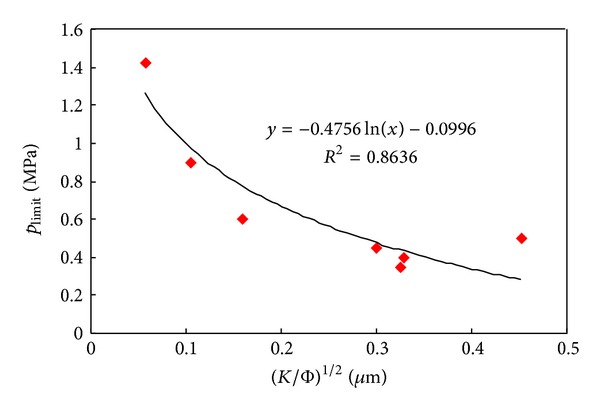
Relationships between integrated logistics index and *p*_limit_ for tight core samples.

*(2) Limit Pressure and Pore Structure*. The experimental results of different permeability indicate that the limit pressure of tight sandstone decreases logarithmically with the increasing in permeability as well as in mean throat radius. The greater the permeability is, the smaller the range of limit pressure will reduce (Table 2, Figures 7 and 8).

For the experimented by Li et al. (2009), the limit pressure was confirmed as 0.68~7.16 MPa by increasing backpressure at outlet of core samples. The Empirical formula is given as [20]

(8) $$\begin{array}{c} p_{\min}=-1.893LnK_{\infty }-2.079. \end{array}$$

**Figure 9:**
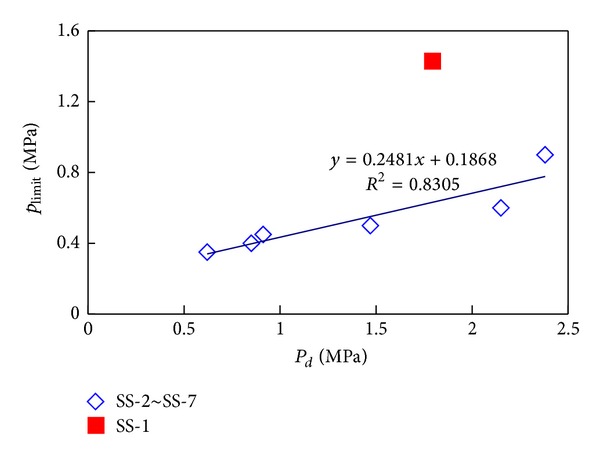
Relationships between displacement pressure and limit pressure for core samples.

The limit pressure from researchers has significant difference as shown in Table 5 [20–23]. The experimental results indicate that limit pressure is 0.35~1.5 MPa. It is close to the results of Zhu et al. (2007) [24] whose experiments also sampled from Permian in Upper Paleozoic in Ordos basin. The test results of this paper are validated by his result. It has been observed experimentally that pore structure has influence on gas slippage. In Figure 8, limit pressure and mean pore throat radius have logarithmic relation. The limit pressure reduced in logarithm with an increase in mean pore throat radius. From Figure 9, limit pressure of tight samples in Ordos basin is directly proportional to displacement pressure, and it is a quarter of displacement pressure. But the relations between limit pressure and displacement pressure are different from the other samples because of diverse pore structures. The limit pressure need quantitative study since it is an approximate value. The relation between pore structure parameters and limit pressure can be developed by fractal theory in porous medium [25, 26]. It is worth caring that, as limit pressure is associated with pore structure, the limit pressure of samples at different area needs to be tested by laboratory experiment.

## 5. Conclusions

*(1) Limit Pressure.* There exists gas slip effect in gas flow through tight sandstone, and exerting a certain backpressure can effectively reduce the gas slip effect. We define this backpressure as limit pressure.

*(2) The Gas Slip Effect Is Negligible.* When the backpressure equals or exceeds limit pressure, the gas permeability tested at different pressure drop is a stable value whose range is less than 3% and slip factor is less than 0.05 for a given sample. The gas slip effect is negligible and the permeability is equivalent to liquid permeability. 

*(3) There Are Close Relationship between the Limit Pressure and Pore Structure.* The limit pressure of tight sandstone decreases logarithmically with the increasing of permeability and mean throat radius and is directly proportional to displacement pressure.

## Figures and Tables

**Table 1:** Basic parameters of core samples.

Samples	*L* (mm)	*D* (mm)	Φ (%)	*K*_*a*_ (10^−3^ *μ*m^2^)
SS-1	57.30	25.10	15.13	0.05
SS-2	77.66	25.02	6.689	0.074
SS-3	59.71	24.74	5.895	0.150
SS-4	63.95	24.73	1.106	0.226
SS-5	59.05	24.76	5.647	0.507
SS-6	62.85	24.73	10.099	1.090
SS-7	59.17	24.59	12.461	1.320

**Table 2:** Basic parameters of core samples.

Samples	*K*_*a*_ (10^−3^ *μ*m^2^)	Φ (%)	*R*_35_ (*μ*m)	*p*_*d*_ (MPa)	*p*_limit_ (MPa)	Relationships between 1/*p*_*m*_ and *K*_*a*_ for samples	*b*	*K*_*a*_ range (%)
SS-1	0.05	15.130	0.023	1.8	1.42	*y* = 0.0001*x* + 0.0032 = 0.0032(1 + 0.031*x*)	0.031	1.83
SS-2	0.074	6.689	0.226	2.38	0.9	*y* = 0.0002*x* + 0.0231 = 0.0231(1 + 0.008*x*)	0.008	0.62
SS-3	0.150	5.895	0.382	2.15	0.6	*y* = 0.0004*x* + 0.0382 = 0.0382(1 + 0.010*x*)	0.010	0.64
SS-4	0.226	1.106	2.061	1.47	0.5	*y* = 0.0006*x* + 0.0771 = 0.0771(1 + 0.008*x*)	0.008	0.60
SS-5	0.507	5.647	0.811	0.912	0.45	*y* = 0.0007*x* + 0.1534 = 0.1534(1 + 0.005*x*)	0.005	2.50
SS-6	1.090	10.099	0.769	0.85	0.4	*y* = 0.0019*x* + 0.4162 = 0.4162(1 + 0.005*x*)	0.005	0.45
SS-7	1.320	12.461	0.718	0.62	0.35	*y* = 0.0108*x* + 0.7911 = 0.7911(1 + 0.013*x*)	0.013	2.06

*R*_35_: mean pore throat radius*; p*_*d*_: displacement pressure; *p*_limit_: limit pressure.

**Table 3:** The influence of backpressure on gas slip factor for sample SS-2.

Backpressure (MPa)	Relationships between 1/*p*_*m*_ and *K*_*a*_ for sample SS-2	*b*
0.1	*y* = 0.0072*x* + 0.0232 = 0.0232(1 + 0.3103*x*)	0.3103
0.3	*y* = 0.0068*x* + 0.023 = 0.023(1 + 0.2957*x*)	0.2957
0.5	*y* = 0.0061*x* + 0.0206 = 0.0206(1 + 0.2961*x*)	0.2961
0.7	*y* = 0.0052*x* + 0.0205 = 0.0205(1 + 0.2537*x*)	0.2537
0.9	*y* = 0.0002*x* + 0.0231 = 0.0231(1 + 0.0086*x*)	0.0086
1	*y* = 0.0001*x* + 0.0227 = 0.0227(1 + 0.0044*x*)	0.0044

**Table 4:** Knudsen number at different pressure for sample SS-3.

*p*_2_ = 0.1 MPa		*p*_2_ = 0.6 MPa	
*p*_*m*_ (MPa)	Kn	*p*_*m*_ (MPa)	Kn
0.35	0.00376	0.85	0.00155
0.625	0.00211	1.1	0.00120
0.85	0.00155	1.35	0.00098
1.1	0.00120	1.6	0.00082
1.35	0.00098	1.85	0.00071

**Table 5:** Experimental results of limit pressure

Time	Author	*K*_*a*_ (10^−3^ *μ*m^2^)	Gas	*p*_limit_ (MPa)
2009	Li et al. [20]	0.0053~0.25	N_2_	0.68~7.16
2007	Zhu et al. [24]	0.01~1	N_2_	0.5
2010	Gao et al. [23]	0.001~2	N_2_	1
2011	Ye et al. [21]	0.024~0.244	N_2_	<7
